# The longitudinal role of early family risks and early social-emotional problems for friendship quality in preadolescence—A regression model

**DOI:** 10.1371/journal.pone.0253888

**Published:** 2021-07-01

**Authors:** Olivia Gasser-Haas, Fabio Sticca, Corina Wustmann Seiler

**Affiliations:** 1 Marie Meierhofer Children’s Institute, Associated Institute of the University of Zurich, Zurich, Switzerland; 2 Zurich University of Teacher Education, Zurich, Switzerland; Victoria University, AUSTRALIA

## Abstract

The quality of a best friendship provides information about how developmentally beneficial it is. However, little is known about possible early risk factors that influence later friendship quality. The present study examined the role of family risks and social-emotional problems (behavioral problems, peer problems, anxious, and depressive symptoms) in early childhood for positive (i.e., support and help) and negative (i.e., conflicts and betrayal) dimensions of friendship quality with their best friend in preadolescence. 293 children (47.9% female) aged 2–4, their parents and teachers participated in the study with three measurement occasions (T1; M_age_ = 2.81, T2; M_age_ = 3.76, T3; M_age_ = 9.69). The last measurement occasion was at the age of 9–11 years. Results of the longitudinal regression model showed that depressive symptoms in early childhood were associated with a lower positive dimension of friendship quality in preadolescence. In contrast, early anxious symptoms were related to a higher positive dimension of friendship quality six years later. Neither family risks, nor behavioral problems and peer problems in early childhood were linked to the positive dimension of friendship quality in preadolescence. No early predictors were found for the negative dimension of friendship quality. Possible reasons for the lack of associations are discussed. Findings suggest that children with early depressive symptoms at 3–5 years of age should be the targets of potential interventions to form high quality friendships in preadolescence. Possible interventions are mentioned.

## Introduction

Besides family relationships, friendships are the most valued and valuable relationships for children, adolescents, and adults alike [1–4]. Although friendships are important for all ages, they become increasingly intimate and influential during preadolescence [5–7]. At this complex transition from childhood to adolescence, children spend more and more time with friends and start to build one-on-one unique high-quality friendships to a single best friend, their first “chumships” [7–10].

At the dyadic-level, there are three types of friendship-adjustment: (1) forming mutual friends, (2) maintaining friendships, and (3) developing high friendship quality from preadolescence onwards [6, 11]. According to Parker and Asher [12], friendship quality is defined as the degree of companionship to the best friend, its supportiveness, and its level of conflict. High-quality best friends are particularly influential on general psychological adjustment. They contribute to children’s happiness, self-esteem, and well-being, help children to cope with challenging situations, and provide a sense of protection [1, 6, 13–15]. Compared to friendships in general or similar relational systems like peer acceptance or the number of friends, high-quality best friendships contribute to higher levels of positive outcomes, even after controlling for individual characteristics [12, 16–18]. A lack of high friendship quality is associated with higher levels of depressive symptoms, peer victimization, and internalizing problems [19, 20].

To summarize, friendship quality is understood as an index of the developmental context that best friendship provides [15]. However, friendship quality varies considerably from friendship to friendship and our understanding of predictors, especially of predictors in early childhood for friendship quality in preadolescence is currently very limited [18, 20–22]. In particular, the effects of risks in early childhood on the later friendship quality have only been marginally investigated [15, 23, 24]. Findings from previous work do indicate that family context and social-emotional skills in early childhood are prerequisites for later peer relationships and the general social functioning of children [20, 24–27]. However, as Schwartz-Mette et al. [24] pointed out, no direct conclusions for the development of best friendship quality can be drawn from this.

For this reason, the aim of the present study was to fill this research gap focusing exclusively on predictors in early childhood as early family risks and early social-emotional problems for friendship quality in preadolescence to determine the extent to which there are associations between problems in early childhood and friendship quality in middle school age.

### Friendship quality—The positive and negative dimension

High-quality friendships are voluntary and reciprocal relationships between two individuals. In contrast to other constructs of peer relationships, as being popular, having a lot of friends, or being accepted in a group, having a close, mutual dyadic relation to a best friend can also exist, if all other peer relation concepts are poor [12, 23–26]. Even children who are unpopular, bullied, or grown up in difficult circumstances can develop high-quality best friendships, but their pool of potential friends is smaller due to their peer rejection or unpopularity [15, 23, 25, 27, 28]. A high friendship quality consists of a positive and a negative dimension, on a continuum [12, 16, 29]. The positive dimension is based on mutual understanding, solidarity, and recognition and is associated with desirable developmental outcomes such as social behavior and self-concept [6, 8, 12, 29]. In contrast, the negative dimension consists of conflicts, rivalry, and a lack of conflict resolution [12, 29, 30] and is linked to undesirable outcomes like loneliness or disruptive behavior [6, 8, 20, 23, 29]. Many studies have focused exclusively on the positive dimension of friendship quality [15]. However, the negative dimension may have a stronger influence on adjustment and wellbeing than the positive one [8, 31], even if findings in this regard are not sufficiently strong [15].

The two dimensions of friendship quality are both important complementary aspects and are only weakly correlated with each other, even though children with a high positive dimension tend to deal better with conflicts than children with a low positive dimension [8, 29, 30, 32]. However, Berndt [29] highlighted that a relationship characterized by a high score on the positive dimension may also have a high score on the negative dimension, too, in particular among older children [33]. Berndt and Peery’s [33] friendship interview findings suggest that for adolescents and adults, a friendship with positive and negative dimensions can coexist, whereas for children, positive and negative dimensions are seen as opposites that are not compatible in the same friendship. Some studies showed that girls reported higher levels on the positive dimension than boys, whereas other researchers did not find differences between boy’s and girl’s [12, 15, 34]. Studies considering the negative dimension reported no gender differences [12, 15, 34, 35].

When it comes to early predictors of the two dimensions of friendship quality, differential associations are possible: While a predictor may exert a positive effect on a given dimension, it may have a negative or negligible effect on the other, or vice versa. Cillessen and Rose [18] showed that adolescents who rated themselves as relationally aggressive perceived their friendships as high in the negative dimension and low in the positive dimension of friendship quality. In contrast, adolescents who saw themselves as prosocial rated their positive dimension as high and their negative dimension as low [18]. According to Bagwell et al. [1, 8], Wise and King [30], and Berndt [29], it is therefore important to consider both dimensions.

The current state of research on friendship quality is based on different versions of questionnaires, short and long versions [15]. In most studies, only a short version of the friendship quality questionnaire was used with a low internal consistency due to the strong reduction of the original instrument [36, 37]. For example, de Lijster et al. [36] shortened the friendship quality questionnaire (FQQ) [12] from 40 to 10 items and achieved an internal consistency of the adapted FQQ of .70 (Cronbach’s alpha). For this reason, it is desirable to use the long version of the friendship quality questionnaire (FQQ) with internal consistency between .73-.90 (Cronbach’s alpha) for each subscale.

### The role of family risks for friendship quality

Wise and King [30] stated that the family, as the primary social environment for an infant, has a significant impact on the child’s future relationships. At best, this effect is positive and can be seen as a resource. The family system supports the child in the development of its self-esteem and self-concept, and helps to build cognitive, emotional, and behavioral schema to interact with others in a socially adequate manner [30]. Moreover, engaged parents initiate play opportunities for their children with other peers to develop prosocial behavior and greater peer acceptance [38]. According to Bowlby [39], a secure attachment can also positively affect the future quality of relationships. Even beyond the period of preadolescence, a strong positive family environment can be an indicator of high quality friendships [30]. However, there are children who grow up in adverse family environments and are exposed to several risk factors. It is widely accepted, that cumulative family risk exposure results in far-reaching undesirable outcomes and is a better predictor for child development than a single indicator [40–44]. Children affected by cumulative family risks are more prone to mental health problems, internalizing and externalizing problems, as well as social relationship problems, ranging from early to middle childhood and adulthood [45–47].

Although current research on the longitudinal impact of early cumulative family risks on the positive and negative dimension of friendship quality in preadolescence is limited [9, 17, 48], several effects of specific family environment factors on friendship quality have already been confirmed. Blair et al. [9] highlighted the positive association between early maternal emotional socialization (parents’ reactions to children’s emotions, parents’ expressions of emotions, and parent-child discussions of emotions) and the positive dimension of friendship quality in middle childhood mediated by children’s emotion regulation. Blair et al. were thus able to show how relevant maternal handling of their own emotions and the emotions in their interaction with their child for friendship quality is. For example, if a mother can respond to the child’s anger and help the child to categorize it, the child is more likely to regulate its own anger, which in turn has a positive effect on friendship quality. Tian et al. [48] pointed out the positive effect of parental support on the positive dimension of friendship quality. Furman [49] reported that secure attachment in parent-child-relationships in infancy is linked to childhood peer competence, which further predicts the positive dimension of friendship quality in adolescence. Furthermore, Laursen and Adams [32] stated that conflicts with family members increase the risk of conflicts with close peers and vice versa.

According to Way and Silverman [20], we know too little about the micro context of the family and about its role for friendship quality. Responding to this research gaps, the present study examined the longitudinal association of cumulative family risks in early childhood on the positive and negative dimension of friendship quality in preadolescence.

### The role of social-emotional problems for friendship quality

Early social-emotional competencies are seen as a prerequisite for later friendship relations and general social behavior [12, 23, 50–52]. For each level of the three types of friendship-adjustment as mentioned before, a certain degree of social and emotional competencies is relevant to finally establish high-quality friendships [6]. Consequently, a lack of social-emotional competencies makes it difficult to build high friendship quality in preadolescence [53, 54].

There is a complex bi-directional interplay between social-emotional competences and friendship relations [55, 56]. On the one hand, researchers emphasized the importance of social-emotional competencies as a prerequisite for later friendships [23, 51], on the other hand, there is evidence that these friendships are important indicators of later social-emotional competencies, adjustment, and well-being [50, 52, 57]. In terms of the friendship quality to a best friend, many studies confirmed the adaptive effect of the positive dimension of friendship quality in (pre-)adolescence on social-emotional competences such as self-efficacy, self-esteem, mental health, and well-being in adulthood [8, 12, 14]. In contrast, only few studies investigated the longitudinal role of social-emotional competences in early childhood, respectively psychological symptoms such as behavioral problems, peer problems, anxious and depressive symptoms in early childhood for friendship quality in preadolescence [36, 58, 59].

#### Behavioral problems

Children with behavioral problems often fight with other children, bully them, are argumentative with adults, and lose their temper [60]. As a result, these children often have difficulty making and maintaining best friendships [1]. According to Dickson et al. [61], externalizing symptoms predict a higher negative dimension and a lower positive dimension of friendship quality in adolescence. One reason for this is the negative downward spiral whereby behavior problems damage friendships, which then may become unstable. Burk and Laursen [62], as well as Kamper and Ostrov [53] stated, that early externalizing problems foreshadow a degradation of later friendship quality, as adolescents with externalizing problems tend to have greater conflicts and less problem-solving skills in their later best-friendship relations [18, 63]. Schneider [23] pointed out, that even the most aggressive child is able to build a best friendship, although they may never reach the point to a high friendship quality, due to the inability to resolve conflicts and to adopt other perspectives. Most studies that have been conducted provide agreement on the negative effects of behavioral problems on friendship quality. However, further studies are needed to specifically describe the effect of behavioral problems from early to middle childhood as the existing studies focus mainly on adolescence.

#### Peer problems

Many researches stated that children who are unpopular, unaccepted, unliked and rejected by peers suffered lifelong adjustment disadvantages and had impairments in friendship relations and future emotional well-being [15, 64]. However, according to Schwartz-Mette et al. [24], over half of the children who had peer problems were able to establish at least one close friendship, even though this friendship was more likely to be lower in quality and less stable over time [2, 15, 56]. Possible reasons for low friendship quality are low social skills and competencies as well as deficits in social information processing, which are directly related to peer problems [12, 15, 28, 56]. Bagwell and Schmidt [15] pointed out that the question of peer problems in general in relation to the quality of friendships is complex. For example, Parker and Asher [12] indicated differences between low- and high-accepted children, as low-accepted children reported less validation, help, and intimate exchange than high-accepted children. Other researchers, such as Lansford et al. [65], failed to find in their distinguishable perceptions of friendship quality in peer problems in their study with fourth-grade-girls, as the rejected girls did not differ in their reported friendship quality compared to the more popular girls. However, observational data showed that rejected girls exhibited less prosocial behavior and social skills in their best friendship compared to average or popular girls [65]. There are much more studies needed to investigate the relation between children’s peer problems and the quality of their best friendships.

#### Anxious and depressive symptoms

Anxious and depressive symptoms play a major role in diminished social competence, peer acceptance, and peer rejection [36, 58, 66, 67]. Although depressive and anxious symptoms are often grouped together as internalizing problems and accordingly examined as a one-dimensional construct, several studies indicated the importance of making a distinction between anxious and depressive symptoms in the association with peer and friendship relations [68].

Children with depressive symptoms are often unhappy, tearful, and worried [60]. Establishing social relationships, especially high friendship quality to a best friend poses great difficulty for children with depressive symptoms [37]. Insofar as children with depressive symptoms have a common and stable friendship, that friend is also often maladjusted himself or herself, has internalizing problems and social-behavioral deficits [1, 23, 69, 70]. Children with depressive symptoms have lower levels of support, stability, help, and intimate exchange in their friendship with a non-adjusted friend [1, 36] and according to Sentse and Laird [71] higher levels of conflict. However, there are mixed results of prediction from depressive symptoms to the negative dimension of friendship quality. Rose et al. [37], for example, found in their study with youth that depressive symptoms negatively predict the positive dimension, but do not predict the negative dimension of friendship quality. While the results regarding the effect of depressive symptoms on the positive dimension are consistent, the effects on the negative dimension are heterogeneous and missing at the age of early childhood into preadolescence.

In contrast to children with behavioral problems and depressive symptoms, children with anxious symptoms are as likely as children without anxious symptoms to make and maintain best friendships, even though their pool of potential mutual friendships is smaller due to their withdrawal from the company of peers [11, 72]. Children with general anxious symptoms are nervous or clingy in new situations, are easily scared and easily lose confidence, but they do desire to play or be with their peers, more so than being alone [11, 58, 60]. In a sample of non-diagnosed third, fifth, seventh, and nine graders, Rose et al. [37] found in their study that general anxious symptoms were a significant positive predictor of the positive dimension of friendship quality six months later, but no significant predictor of the negative dimension. In contrast, socially anxious and withdrawn children are associated with friendship problems and a lower positive dimension of friendship quality [72]. However, Rodebaugh et al. [57] concluded in a sample of young adults, aged 17–22 years, that social anxiety did not predict the positive dimension of friendship quality. According to Rubin et al. [72], it is crucial to investigate the effects of anxious symptoms to friendship quality across different periods of development, as it is assumed that these effects change with age. The effects of anxious symptoms in early childhood have hardly been investigated to date on the positive and specifically on the negative dimension of friendship quality in preadolescence for non-diagnosed children.

### Research questions and hypotheses

The aim of the study was to address previous limitations by examining the impact of cumulative family risks as well as social-emotional problems in early childhood on the positive and negative dimension of friendship quality in preadolescence. We hypothesized that (H1a) early family risks (T1) would be negatively associated with the positive dimension and (H1b) positively associated with the negative dimension of friendship quality in preadolescence (T3). Using a multi-informant approach, which according to Kraemer et al. [73] has proven fruitful in early childhood, we examined the effects of social-emotional problems (T2) on positive and negative dimensions of friendship quality in preadolescence (T3). We expected that (H2a) behavioral problems would be negatively linked to the positive and (H2b) positively linked to the negative dimension of friendship quality. Moreover, we hypothesized that (H3) depressive symptoms would be negatively related with the positive dimension of friendship quality. Finally, we assumed that (H4) anxious symptoms would be positively associated with the positive dimension of friendship quality. Due to the limited and heterogeneous data, no hypotheses for the associations of anxious and depressive symptoms and the negative dimension of friendship quality as well as of peer problems and the positive and negative dimension of friendship quality were formulated.

## Method

### Participants

The longitudinal study from early childhood to preadolescence encompassed three measurement occasions and was part of the project “Long-term effects of early family risk on children’s maladjustment and self-efficacy: individual, familial and extra-familial protective processes” (2016–2019) that belongs to the project “Promoting early learning and resilience through a strengthening learning dialogue–A project for promotion and professionalization of early childhood education in Swiss childcare centers” (2009–2012). At the start of the study, in 2009, 293 children and their parents were recruited from 25 childcare centers in Switzerland and interviewed (T1; 47.9% female, M_age_ = 2.81, SD_age_ = 0.55). One year later, at the second measurement occasion, 239 of the total 293 children (T2; 47.3% female, M_age_ = 3.76; SD_age_ = 0.49) participated in the study. Finally, in 2016, 189 of the same children, now aged nine to eleven years, took part in the long-term follow-up study six years later (T3; 48.6% female, M_age_ = 9.69, SD_age_ = 0.48). Of the total of 293 families, most were Swiss origin, German speaking, and from the upper-middle class: 62% of mothers and 64% of fathers had a university degree at T1, six years later at T3 70% of the mothers and 74% of the fathers had graduated. 16% of the children had a foreign language background at the beginning of the study.

The number of dropouts between T1 and T3 was 103 children. Though, the retention rates were relatively high, considering that there are around seven years. The analyses of the missingness over the three times of measurement showed that children who participated in both T1 and T2 had marginally lower overall scores of family risks (ß = -.09; *p* = .09) than children who participated just in T1. However, the difference was not significant. The same pattern was found for the drop out from T2 to T3. Children participating in all three times of measurement had marginally but not significantly lower scores of family risks (ß = -.13; *p* = .06) than the children who participated just in T1 and T2. Children who participated in both, T2 and T3 had comparable scores of behavioral and emotional problems as children who participated just in T2 (behavioral (ß = -.02; *p* = .69) and emotional problems (ß = -.05; *p* = .71)).

### Procedure

The recruitment was organized via childcare centers. Parents received a letter containing the study description and the declaration of consent. Teachers obtained these documents at the second and children at the third measurement occasion. Children, parents, and teachers agreed to participate and permitted an interview and/or to complete a questionnaire. All parents gave their written informed consent and were informed of their right to quit their participation at any time without indicating any reasons. Trained research assistants carried out the parent and child interviews and administered the questionnaires. All participating persons were informed that data would be anonymized after completion of the data collection and be used for research purposes only. Children received a small gift after each assessment, such as a book voucher at T3.

All procedures performed in the study were in line with the Swiss legislator for research with human participants and the Declaration of Helsinki 1964 and its later amendments. As the local ethics committee of the Swiss Ethics Committees for Research on Human Beings declared, at none of the three measurement occasions ethical approval was required.

### Study measures

#### Family risks

Family risks were assessed by a parent interview for the first time of measurement. One part of the questions was administered during the interview. The other part, which contained more delicate questions, was administered using a paper and pencil questionnaire. The items of the questionnaire and the interview were composed of various validated instruments previously used in research on family risks according to a standardized procedure [e.g., 74, 75]. We subsumed the following 14 dichotomous or dichotomized psychosocial and socioemotional family risks into one cumulative overall family risks score (*M* = .08; *SD* = .08): (1) family income below the poverty threshold (12.7%), (2) immigrant background of the family (16.8%), (3) current and/or previous chronic partnership disharmony (10.7%), (4) single parent family (10.0%), (5) low maternal education (7.9%), (6) alcohol and/or drug abuse of father and mother (5.4%), (7) self-reported mental health issues of mother and/or father (4.9%), (8) serious illness of a sibling (4.1%), (9) current and/or previous family violence (3.4%), (10) serious illness or death of a primary caregiver (3.0%), (11) serious illness or death of another family member (2.7%), (12) current or previous issues with the law (1.9%), (13) serious illness or death of a friend (1.0%), and finally (14) family move (25.0%).

#### Social-emotional problems

In the second measurement occasion, parents and teachers completed the Strengths and Difficulties Questionnaire (SDQ) [60] for a multi-informant approach to obtain adequate, reliable and valid measures of behavioral and emotional problems in early childhood [73, 76]. The SDQ is widely used and well established to identify the strengths and difficulties of socio-emotional competencies of 3-16-year-olds. It consists of five subscales: peer problems, emotional problems, conduct problems, hyperactivity, and prosocial behavior, each with five items. The items are rated on a 3-point Likert scale from 1 (not true) to 3 (certainly true). In the present study, we used the following subscales: peer problems, conduct problems (behavioral problems), and emotional problems. To generate the multi-informant approach, the items assessed by parents and teachers were averaged. A confirmatory factor analysis was carried out of the subscale behavioral problems. Due to cross loadings of two items and an insufficient model fit (χ2[5] = 10.96; CFI = .97; RMSEA = .08; SRMR = .03), one items was eliminated, which led to a final model fit of χ2[2] = 1.86; CFI = 1.00; RMSEA = .001; SRMR = .01 with a McDonald’s Omega of .77. For peer problems, a confirmatory factor analysis showed that one item had a low factor loading of .22, which was then removed from the scale. According to Hair [77] and Hulland [78], it is recommended that items with an amount of explained variance of less than 25% be removed from a scale because they have little explanatory power for the model. The subscale of peer poblems reached a McDonald’s Omega of .65 in the multi-informant approach. Given the just acceptable reliability, the peer problems were modeled as a latent variable to remove measurement error and situational effects [79]. The original subscale of emotional problems was separated into depressive and anxious symptoms for theoretical reasons consisting of two items: Depressive symptoms (“Often unhappy, down-hearted or tearful” and “Many worries or often seems worried“) and anxious symptoms (“Nervous or clingy in new situations, easily loses confidence” and “Many fears, easily scared”). One item was excluded because the content of this item could not be clearly assigned to either depressive or anxious symptoms, as evidenced by the low factor loading on both anxious (.18) and depressive (.31) symptoms. McDonald’s Omega reliability values were .74 for anxious and .82 for depressive symptoms in the multi-informant approach of parents and teachers.

#### Friendship quality

To assess children’s best friendship quality in preadolescence (T3), the long version of the well-established Friendship Quality Questionnaire (FQQ) [12] was used. Children were asked to report their best friend’s name first to make a clear reference. 87.1% of all children were able to name a best friend. The questionnaire consists of 40 items with six subscales, five positive components, and one negative component. Each item of the components was rated on a 5-point Likert scale from 1 (not at all true) to 4 (absolutely true). The children rated the items of four positive components by themselves to assess their *positive dimension* of friendship quality concerning their best friend: Help and Guidance (e.g., “Do special favors for each other”), Intimate Exchange (e.g., “Talk about the things that make us sad”), Validation and Caring (e.g., “Makes me feel good about my ideas”), and Conflict Resolution (e.g., “Make up easily when we have a fight”). The positive component “Companionship and Recreation” was not used in the present study, as the items required that the children attend school together (e.g., “Always play together at recess”), which was not the case. All items of the four positive components, Help and Guidance (Mc Donalds’s Omega = .85), Intimate Exchange (Mc Donald’s Omega = .88), Conflict Resolution (McDonald’s Omega = .74), and Validation and Caring (McDonald’s Omega = .87) were used as in the original instrument. The four components were modeled as manifest indicators of the latent positive friendship quality with a final McDonald’s Omega of .91. To assess their self-perceived *negative dimension* of friendship quality, the children estimated the items of the negative component: Conflict and Betrayal (e.g., „Get mad a lot“). Due to low factor loading of .20, and a weak model fit (*χ2*[14] = 28.61; CFI = .91; RMSEA = .08; SRMR = .06), one items was eliminated, which led to an accepted model fit of *χ2*[9] = 13.08; CFI = .97; RMSEA = .05; SRMR = .05 with a McDonald’s Omega of .90. The negative component of friendship quality was modeled manifestly.

### Analysis strategy

**Fig 1:**
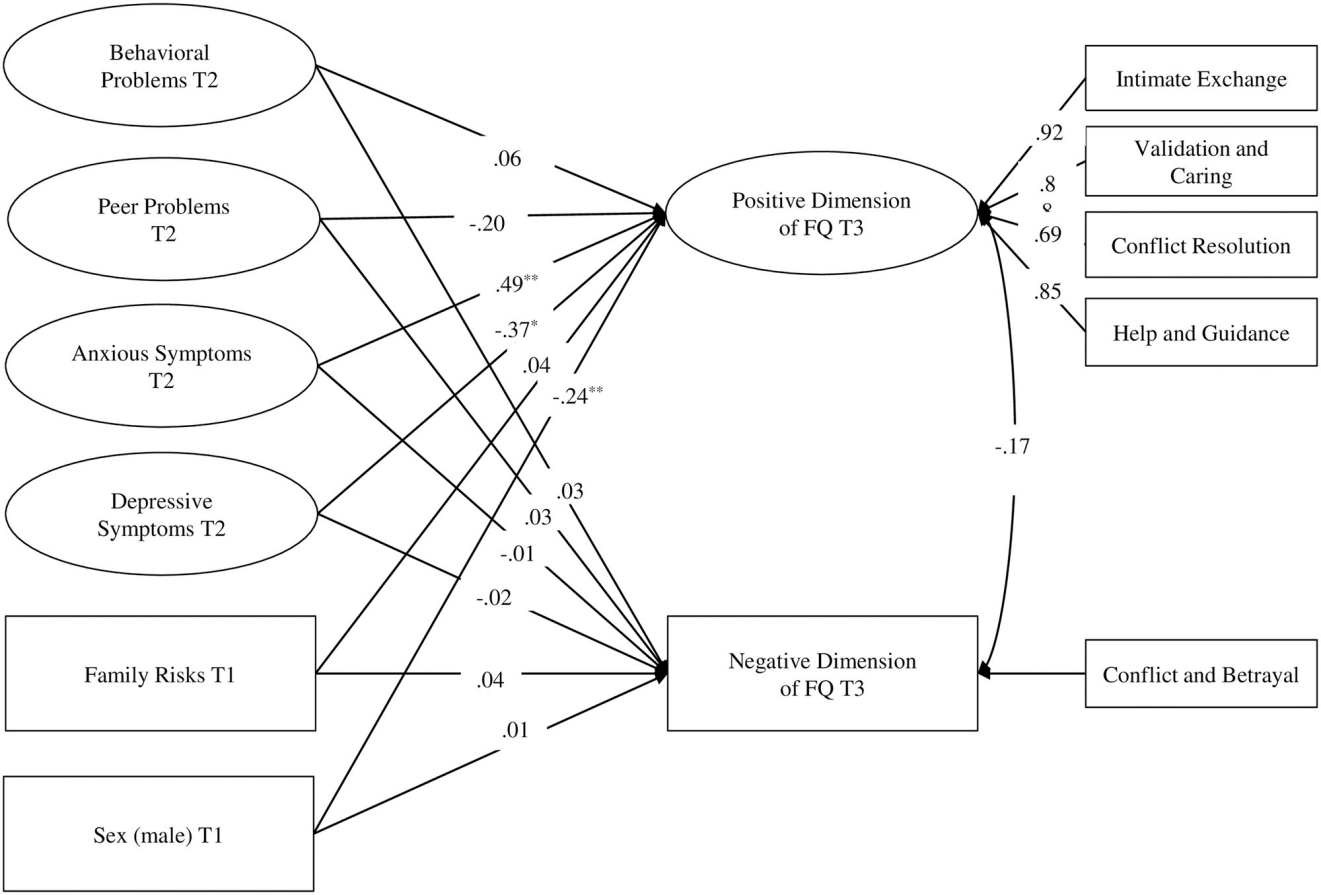
Standardized results from the model. T1-T2-T3 = Data measurement occasions. Model fit (*χ2*[110] = 136.60; CFI = .97; RMSEA = .03; SRMR = .05). *p < .05; **p < .01.

To analyze the association of family risks, behavioral problems, anxious and depressive symptoms, and the positive as well as the negative dimension of friendship quality, we conducted a full structural equating model (SEM), using Mplus Version 8.0 [80] (Fig 1).

A single model was created to examine all hypotheses. Herein, two dependent variables were modeled, namely the positive and the negative dimension of friendship quality. While the negative dimension consisted of a single subscale and was thus modeled as a manifest variable, the positive dimension contained four subscales that were modeled as indicators of a latent variable representing the positive dimension of friendship quality. Following the suggestions by Little [81], the latent variables were modeled with the effect coding method. Behavioral problems, peer problems, anxious and depressive symptoms were introduced as latent predictors of both the positive and negative dimension of friendship quality. Moreover, family risks were included as a manifest predictor variable and children’s sex as a control variable. Finally, all predictors and the control variable were allowed to correlate with each other. The model fitted the data well (*χ2*[110] = 136.60; CFI = .97; RMSEA = .03; SRMR = .05). The full information maximum likelihood (FIML) for missing data was applied [82].

## Results

### Bivariate correlations among all study variables

Table 1 shows intercorrelations (zero-order correlations) between all study variables as well as their means and standard deviations.

**Table 1:** Bivariate correlations among all study variables (n = 293).

		M	SD	1	2	3	4	5	6	7
1	Positive Dimension of FQ T3	2.86	0.73	1						
2	Negative Dimension of FQ T3	0.42	0.48	-.16	1					
3	Family Risks T1	0.08	0.50	.05	.10	1				
4	Behavioral Problems T2	1.41	0.29	-.04	.04	.26**	1			
5	Peer Problems T2	1.29	0.21	-.18	.03	.15	.08	1		
6	Anxious Symptoms T2	1.43	0.32	.15	-.01	.18*	.04	.44***	1	
7	Depressive Symptoms T2	1.14	0.23	-.14	-.01	.26**	.15	.45***	.66***	1
8	Sex (male) T1	1.52	0.08	-.24**	-.01	-.01	.24**	.13	.08	.01

FQ = Friendship Quality; T1-T2-T3 = Data measurement occasions.

*p < .05;

** *p* < .01;

***p < .001.

Family risks were positively correlated with behavioral problems, depressive symptoms, and anxious symptoms. Anxious symptoms and depressive symptoms were positively correlated with each other and positively correlated with peer problems. Further, boys had significantly more behavioral problems than girls. Girls had significantly higher scores on the positive dimension than boys.

### Results from the structural equation model

Regarding the role of early family risks, there was no association with the positive dimension of friendship quality (ß = .04, *p* = .59). At the same time, no association with the negative dimension of friendship quality could be found (ß = .04, *p* = .60).

As for social-emotional problems, behavioral problems were unrelated to the positive dimension (ß = .06, *p* = .55), as well as the negative dimension of friendship quality (ß = .03, *p* = .78). Peer problems were marginally negatively associated with the positive dimension (ß = -.20, *p* = .10), and unrelated to the negative dimension of friendship quality (ß = .03, *p* = .80). Depressive symptoms at T2 were significantly negatively associated to the positive dimension of friendship quality at T3 (ß = -.37, *p* < .05), as children with higher scores on depressive symptoms at T2 tended to have a lower positive dimension of friendship quality six years later. In contrast, depressive symptoms at T2 were unrelated to the negative dimension of friendship quality at T3 (ß = -.02, *p* = .90), implying that children with depressive symptoms have the same level of the negative dimension of friendship quality as children without depressive symptoms. As for the prediction of the positive dimension of friendship quality in preadolescence, early anxious symptoms were found to be related medium to large, and significant (ß = .39, *p* < .05). Anxious symptoms were not associated with the negative dimension of friendship quality six years later (ß = .05, *p* = .73).

Finally, results regarding the role of the covariates suggested that girls tended to have higher scores on the positive dimension of friendship quality than boys (ß = -.24, *p* < .01). In contrast, no difference could be found between girls and boys on the negative dimension (ß = .01, *p* = .96).

## Discussion

The present study examined the role of early family risks as well as early social-emotional problems for the positive and the negative dimension of friendship quality in preadolescence. Results are discussed starting from the role of family risks, to the role of social-emotional problems (behavioral problems, peer problems, anxious, and depressive symptoms), and finally to the lack of associations with the negative dimension of friendship quality in preadolescence.

### The role of family risks

No relevant association of early family risks with the positive dimension of friendship quality in preadolescence was found, which led to the rejection of hypothesis 1a. Children with early family risks seem to have the same chance to build a positive dimension of friendship quality in preadolescence as children without family risks. One explanation can be seen in the fact that children who experience a low level of positive parenthood spend a large part of their free time with friends, which might be conducive to the development of the positive dimension of friendship quality. Another point may be that children can have a good parent-child relationship despite family risks, in the sense that children’s attachment to their parents has a positive influence on the children’s positive dimension of friendship quality regardless of family risks, however, this hypothesis would need to be verified. At the same time, children with early family risks tend to have the same level of conflicts in their best friendship like children without family risks, which leads to the rejection of hypothesis 1b. In contrast to previous research [30, 32], results showed no effect of early family risks on the negative dimension of friendship quality in preadolescence.

### The role of behavioral problems

We found negligible effects of behavioral problems in early childhood on the positive and negative dimension of friendship quality in preadolescence and both hypotheses 2a and 2b had to be rejected. Children with behavioral problems do not seem to differ from children without behavioral problems in their positive and negative dimension of friendship quality. One explanation could be that many young children succeed in overcoming their behavioral problems in middle childhood [83, 84], which would consequently have a positive effect on the positive dimension of friendship quality. Further research is needed at this point.

Besides, children with non-developmentally-related behavior problems are more likely to overestimate themselves in general and perhaps also in terms of their friendship quality, which leads to an underestimation of the effects [85, 86]. However, the most likely explanation is based on the principle of homophily [23]. Friends resemble themselves and are similar in a number of aspects, like sex, age, race, and social status but also in interests, tastes, attitudes, and characteristics [87]. Therefore, it is possible that children with behavioral problems tend to form best friendships with other children who have behavioral problems themselves. According to Schneider [23], homophily or similarity is not always a good thing. If friends with behavioral problems have a high friendship quality, it is assumed that they have an increased risk of bullying or being argumentative with adults and children outside of their best friendships and exacerbate their behavioral problems.

### The role of peer problems

Children with peer problems in early childhood slightly tend to have a lower positive dimension of friendship quality six years later. In line with Parker and Asher [56] as well as Bagwell and Schmidt [15], children with peer problems in early childhood have a decreased probability of forming later high-quality friendships, because of lower social skills and social information processing deficits. Although it is still assumed that different competencies are needed to succeed at the peer level and at the level of establishing best high-quality friendships [15], there seems to be a transfer between the group level of peer problems and the quality of best friendship.

In contrast, children with early peer problems had the same level of the negative dimension of friendship quality in preadolescence as children without peer problems. This may be because children with peer problems often view conflicts as joking and fun, even though their friends are more likely to view it as immature and problematic [11, 88]. In addition, these children may be limited in their perception and assessment of friendship quality by a lack of experience of high-quality friendships [11].

### The role of depressive symptoms

Results showed that children with depressive symptoms at the age of three to five years tend to have lower scores on the positive dimension of friendship quality six years later, which leads to the confirmation of hypothesis 3. Nangle et al. [28] as well as Pössel and Hautzinger [67] highlighted the negative relationship between depressive symptoms and the positive dimension of friendship quality, even though the present study was the first to demonstrate the relationship from early childhood into preadolescence.

In contrast to the positive dimension of friendship quality, the current study found no meaningful effect between depressive symptoms and later friendship quality. While for example Schwartz-Mette et al. [24] found an overall correlation coefficient of .18 regarding the association of depressive symptoms with later negative dimension of friendship quality in their meta-analysis, Rose et al. [37] found no meaningful effect between depressive symptoms and later friendship quality. The results of the study support the findings of Rose et al. [37] in ages from early childhood to preadolescence.

### The role of anxious symptoms

The results of our study indicate that anxious symptoms at the age of three to five years were linked to higher scores on the positive dimension of friendship quality in preadolescence. Hypothesis 4 is thus supported. While Rose et al. [37] could show that anxious children had a higher positive friendship quality half a year later in their study with non-diagnosed third, fifth, seventh, and ninth graders, our results underpin that this pattern also applies to children from early childhood to preadolescence, with an interval of six years. Especially for one-on-one high-quality friendships, anxious symptoms are promotive. While children with anxiety symptoms might find themselves to be uncomfortable in a larger peer group, they could feel socially secure, emotionally supported, and satisfied in their best friendship [11]. Once they have built the best friendship, they will tend to invest as much as possible in that relationship and thus lay the first stone to develop a high-quality best friendship out of it.

These results contrast to children with social anxiety or diagnosable anxiety disorders [11, 37]. These children might tend to score lower on the positive dimensions of friendship quality due to withdrawal in general social situations, including social interactions with a best friend. This withdrawal may lead to deficits in social skills due to lack of practice, as well as an inability to provide social support in a relationship.

In line with Rose et al. [37], we found just a negligible effect of anxious symptoms on the negative dimension of friendship quality. In contrast to children with depressive symptoms, children with early anxious symptoms have a sufficiently high level of self-esteem, self-worth, and self-efficacy to address conflicts in a relationship and defend themselves [22, 36]. Thus, these children might experience normal level of conflicts in the relation with their best friends.

### Lack of associations for the negative dimension

The present results suggest that early social-emotional problems are linked to the positive dimension of friendship quality. In contrast, no associations with the negative dimension were found. On the one hand, this could be due to the fact that the two dimensions of friendship quality are important complementary aspects [e.g., 29], suggesting that other predictors may be relevant and need to be considered accordingly or that for children at this age a coexistence of a positive and negative dimension of friendship quality is not compatible [e.g., 33]. On the other hand, according to Bagwell and Schmidt [15], conflicts must always be seen in the context of the positive dimension of friendship quality: High levels of conflicts might be less problematic in the presence of a high score on the positive dimensions of friendship quality. In contrast, conflicts may lead to problematic adjustment when coupled with low scores on the positive dimensions of friendship quality. Therefore, further research is needed to identify relevant predictors in early childhood for the negative dimension of friendship quality in preadolescence.

### Strengths and limitations

The strengths of the present study lie in the expansion of the existing knowledge of friendship research by examining predictors in early childhood for friendship quality in preadolescence. Many researchers have argued for the developmental significance of friendship quality [1, 6, 13, 14, 29], while relatively little is known about the variables that predict positive and negative dimensions of friendship quality. Especially, variables in early childhood from at-risks samples are still understudied. Moreover, to the best of our knowledge, this study is the first to longitudinally examine the association of cumulative family risks, behavioral problems, and peer problems in early childhood on later friendship quality. Furthermore, in contrast to previous research, we used a long version of the questionnaire on friendship quality from Parker and Asher [12], which allowed us to achieve a higher internal consistency.

The study also encountered some limitations. The preadolescent children assessed their friendship quality by themselves. Although the most common approach to measure the quality of friendships is through children’s self-reports [15], we could neither test whether the best friendship was based on reciprocity nor if they agree on their quality of friendship. According to Bagwell et al. [8], disagreement between friends about the quality of their friendship is associated with individual maladjustment and can, therefore, be regarded as an important factor to consider in a study on friendship quality. Finally, no cause-and-effect relationships could be made based on the study design.

### Implications for practice

Results indicate that children with depressive symptoms in early childhood are particularly affected by later impairments in friendship quality with their best friend. Often, children with depressive symptoms exhibit negative moods and themes, a lack of appropriate social skills, and a negative appraisal of their relationship with others and with their best friends, which can negatively impact friendship quality. Therefore, it is important to support and encourage children with early depressive symptoms to participate in social interactions and help them improve their interpersonal interaction behaviors. The following suggestions are relevant for parents, child-care institutions, and schools. Social skills training and social cognitive interventions show promise in teaching specific skills for forming shared friendships, maintaining friendships, and developing high-quality friendships. For example, Asher and colleagues [89] identified 10 specific social skills for successfully forming friendships. Especially for younger children, parental involvement should be included in intervention efforts. Parents of young children are important players when it comes to building friendships because of their influence on children’s social opportunities. At best, interventions should actively allow children to be in direct interaction with other children, such as small group interventions or whole class interventions. At most, it may be necessary to clarify whether children may need therapeutic support regarding their depressive symptoms.

## Conclusion

The present results suggest that children with increased family risks in early childhood have normal odds of forming best friendship quality. In contrast, children with early depressive symptoms tend to be at higher risk of developing a low positive dimension of friendship quality in preadolescence. Therefore, child-care institutions and schools should be aware of young children with early depressive symptoms. The affected children need support in their relationships with peers, to strengthen their positive interactions, to improve their social skills, and to finally enable them to build high-quality friendships. Further longitudinal research with large samples is required to investigate the direction of causality and to identify relevant predictors in early childhood for the friendship quality in preadolescence.

## Supporting information

S1 Dataset Dataset used in the study. (CSV)
